# School absence and mental health service referral: Cohort study of a South London data linkage

**DOI:** 10.1002/jcv2.70144

**Published:** 2026-07-09

**Authors:** Sophie Epstein, Zoë Firth, Alice Wickersham, Hitesh Shetty, Amelia Jewell, Rina Dutta, Craig Morgan, Tamsin Ford, Richard D. Hayes, Johnny Downs

**Affiliations:** ^1^ Department of Child and Adolescent Psychiatry CAMHS Digital Lab King's Maudsley Partnership Institute of Psychiatry, Psychology & Neuroscience King's College London London UK; ^2^ South London and Maudsley NHS Foundation Trust London UK; ^3^ Centre for Academic Mental Health, Population Health Sciences Bristol Medical School, University of Bristol Bristol UK; ^4^ NIHR Maudsley Biomedical Research Centre London UK; ^5^ Department of Psychological Medicine Institute of Psychiatry, Psychology and Neuroscience King's College London London UK; ^6^ ESRC Centre for Society and Mental Health King's College London London UK; ^7^ Department of Health Service and Population Research Institute of Psychiatry, Psychology and Neuroscience King's College London London UK; ^8^ Department of Psychiatry University of Cambridge Cambridge UK

**Keywords:** longitudinal study, mental health, school absence

## Abstract

**Background:**

The longitudinal relationship between school absence and mental health has important policy implications; if school absence predicts later mental health problems, it could be used to identify young people at increased risk and enable further assessment, prevention, and early intervention.

**Methods:**

We analysed an existing data linkage between the National Pupil Database and healthcare records representing a sample of 47,926 young people aged 11–15 in the UK. We used logistic regression to examine the longitudinal association between persistent school absence (defined by the Government Department for Education in England as missing more than 10% of available school sessions) and later contact with secondary care mental health services. We also compared the sensitivity and positive predictive value of the >10% absence threshold to alternative thresholds for predicting adverse mental health outcomes.

**Results:**

At the currently applied threshold of >10%, persistent school absence was associated with 2.77 (95% CI 2.33–3.30, girls) and 1.58 (95% CI 1.29–1.95, boys) times the odds of accepted referral to secondary care mental health services in the following year, after adjustment for sociodemographic and educational factors. The absolute risk difference for girls was 4.7% (95% CI 3.9–5.4) and for boys, 2.8% (95% CI 2.1–3.4). Compared to other thresholds, the >10% absence threshold provided a good balance between sensitivity (40.5%), positive predictive value (5.5%), and identifying a manageable proportion of young people as high risk (18%).

**Conclusion:**

The longitudinal relationship between school absence and later secondary care mental health service contact suggests that school absence may be a useful marker for educators to identify children in need of support. The >10% absence threshold used in education policy in England may also serve as a useful marker of later mental health risk in other national policy contexts.

## Key Points

**What's known?**

- There is an established cross‐sectional association between young people's absence from school and their mental health.

**What's new?**

- Less is known about whether school absence predicts later mental health.
- Here, we tested the longitudinal relationship between school absence and later mental health (accepted referrals to secondary care child and adolescent mental health services) amongst secondary school children in the UK, utilising a data linkage comprising over 40,000 pupils.
- A school absence level of more than 10% predicted the likelihood of having a mental health service referral accepted within the following year.

**What's relevant?**

- These findings suggest that high levels of school absence could be used to help identify young people at risk of mental health problems and provide assessment, prevention, and early intervention, insights which may help to formulate effective health and education policy.

## INTRODUCTION

School absence is associated with a range of adverse outcomes including poorer academic attainment (Smerillo et al., 2018) and higher levels of violence, injury, and substance misuse (Egger et al., 2003). There is evidence that school absence is associated with poor mental health (Fleming et al., 2019; John et al., 2022), indicating that school absence could be used as a marker to identify young people at higher risk. This could in turn enable early intervention that may prevent or mitigate negative mental health and educational outcomes. School absence has been an increased concern across the world since the COVID‐19 pandemic (Dee, 2024), suggesting the presence of unmet needs which need to be addressed through both health and education policy and practice (Shankar et al., 2024).

While a variety of cross‐sectional studies in North America, Europe, and Asia have found a relationship between school absence and worse mental health (Finning et al., 2019a, 2019b), fewer have examined the longitudinal relationship between school absence and later poor mental health outcomes (Epstein, 2024). This research has produced initial evidence—albeit with mixed findings and varied designs—for a longitudinal relationship between school absence and mental health (Krause et al., 2025; Panayiotou et al., 2023; Wood et al., 2012), which is likely also bidirectional (Ford et al., 2018). This relationship is likely to be influenced by a variety of clinical and sociodemographic factors which are known to be associated with both school absence and mental health problems (Reiss, 2013), for example Special Educational Needs (SEN) (Núñez et al., 2025; Tanya Lereya et al., 2023), exclusion from school (Ford et al., 2018; John et al., 2022; Tejerina‐Arreal et al., 2020), age and socio‐economic status (Sosu et al., 2021).

Many previous studies used self‐report data on absence, increasing the risk of recall or reporting bias (Keppens et al., 2019), or did not evaluate which absence threshold is most effective at predicting risk, thus making findings less informative for policy. At a policy level, an absence threshold which is a useful marker of underlying risk will identify a manageable proportion of the student population as high‐risk to be offered further assessment, support, and preventative intervention. The threshold should be sufficiently sensitive (i.e., the threshold identifies a sufficient proportion of those who do go on to develop mental health problems), while not generating too many false positives (i.e., of those who meet the absence threshold, how many do in fact go on to develop mental health problems). In England, the Department for Education (DfE) defines a threshold for ‘persistent absence’ as missing >10% of available half‐day school sessions across an academic year. The >10% threshold has also been employed as a definition of ‘chronic absenteeism’ in research and policy elsewhere (e.g., Kirksey, 2019).

In this study, we used routinely collected school absence data linked to clinical mental health data in South London to investigate the longitudinal association between school absence at a >10% threshold, and later severe mental health problems, as operationalised by having a referral accepted by secondary care mental health services. We also compared the utility of England's DfE >10% absence threshold with other thresholds for identifying young people at higher risk of later severe mental health problems.

## MATERIALS AND METHODS

### Cohort and study design

This is a retrospective cohort study, where we used an existing linkage between electronic healthcare records (the Clinical Records Interactive Search) and an educational administrative dataset (the National Pupil Database).

‐ **The Clinical Records Interactive Search (CRIS) database** is a secure de‐identified database of electronic health records for all patients who have been referred to secondary care mental health services at South London and Maudsley NHS Foundation Trust (SLaM) since 2007 (Stewart et al., 2009). SLaM is a public provider of mental healthcare, covering four boroughs of South East London with a combined population over 1 million (Perera et al., 2016). The SLaM catchment area has a higher proportion of people from ethnic minorities and low socioeconomic groups than the UK average (Perera et al., 2016).
‐ **The National Pupil Database (NPD)** is a dataset held by the DfE in England covering pupils enrolled at all state schools, comprising data routinely collected by schools.

The linkage between CRIS and the NPD comprised a cohort of young people aged 4–17 inclusive who were referred to Child and Adolescent Mental Health Services (CAMHS) at SLaM between 1st September 2007 and 31st December 2013. To achieve the linkage, personal identifiers of first name, surname, date of birth, and postcode were provided by SLaM to the DfE along with the CRIS unique patient identifier. These records were then matched by the DfE to NPD records through a process of deterministic linkage using exact and fuzzy matching on name, date of birth, and postcode in several stages. Details of the linkage process, ethics, and governance are described elsewhere (Downs et al., 2019). Of the whole population sample of 230,000 young people represented in the NPD and living in the SLaM catchment area, 35,509 individuals had eligible records in CRIS (Downs et al., 2019). Of these, it was possible to match the CRIS and NPD records of 29,278 individuals (82%). The possibility of linkage bias, wherein certain groups are more likely to match records than others (Gilbert et al., 2018), has previously been investigated in this data linkage. While some socio‐demographic variables, including ethnicity, were found to be associated with the likelihood of matching records, the relationship between school absence and mental health remained after using statistical techniques to adjust for matching probability (Downs et al., 2019), hence the effect of linkage bias on the relationship between these two variables here is likely minimal.

### Eligibility for this study

Of the whole population sample of 230,000 young people represented in the NPD and living in the SLaM catchment area, young people were eligible for inclusion in this study if they were aged between 11 and 15 years at the start of the 2010/11 academic year and resided in one of the four boroughs of the SLaM catchment area during the 2010/11 academic year.

### Variables

#### Exposure variable

The exposure of interest was total absence (whether authorised or unauthorised) during the 2010/11 academic year, measured from the start of the autumn term (on or around 1st September 2010) up to the end of May 2011. School absence data is coded according to the number of half‐day sessions missed as a proportion of sessions available to each pupil on a termly and annual basis (Department for Education, 2015). Until 2012, absence data represented only five of the six half terms of the academic year; the sixth half term was considered not to be representative of overall absence patterns, as many pupils take study leave at this time to prepare for final examinations. Here, absence was therefore calculated using only data from the first five half terms.

For the analysis of the utility of different thresholds of high absence, absence was converted into a binary measure at the following thresholds of sessions missed: >1%, >5%, >10%, >20%, >30%, >40%, and >50%.

#### Outcome variable

The outcome was operationalised as having an accepted referral to CAMHS. This was defined as having a referral accepted by SLaM CAMHS where the referral date fell between 1^st^ June 2011 and 31^st^ May 2012 that is, during the 1 year following the end of the exposure period. Young people whose referrals to CAMHS were rejected or who had episodes of CAMHS care which began prior to the start of the follow‐up period were therefore coded as having no accepted referral during the follow‐up period.

#### Covariates

All data on covariates were taken from the NPD for the 2010/11 academic year.

#### Sex/gender

A binary variable, boy and girl. We acknowledge that sex and gender are different constructs, and not binary (Heidari et al., 2016), however the data are collected as male/female only, and when collected, do not specify whether this refers to sex or gender. Hence, this variable may refer to either sex and/or gender depending on the interpretation of the individual who supplied the information.

#### Age at start of academic year

A continuous variable, with years of age 11–15 inclusive, which serves as a proxy for school year group.

#### Free School Meals eligibility

A binary variable (yes/no) which serves as a proxy for a pupil's socioeconomic status, as eligibility for Free School Meals (FSM) is means‐tested.

#### Special educational needs provision status

A binary variable, based on the four‐category variable coded by the SEN provision codes in the NPD at the time, written here in increasing order of need: None, School Action, School Action Plus, and Statement (Department for Education & Department of Health, 2015). Here, the binary variable for SEN provision (yes/no) was generated by grouping School Action, School Action Plus, and Statement together.

#### Exclusion

A binary variable representing whether a young person was excluded from school (permanent or fixed term) at any point during the 2010/11 academic year before 31^st^ May 2011. Referred to elsewhere as ‘suspension’ (i.e., fixed term exclusion) or ‘expulsion’ (i.e., permanent exclusion), exclusion is typically an enforced removal from school as a disciplinary measure (Department for Education, 2024a).

#### Summer birth

A binary variable calculated using birth month, wherein individuals born between May and August inclusive were considered summer born. Summer births are associated with certain mental health problems (Goodman et al., 2003; Root et al., 2019), a finding attributed to the fact that those with summer births are among the youngest in a school year.

### Statistical analysis

All analyses were conducted using Stata MP 15 for Windows. First, missing data and the representativeness of the analysis sample were examined. Descriptive statistics for absence were generated for the whole sample, stratified by sex/gender and each of the covariates.

The main logistic regression analyses examining the association between school absence and later accepted referral to CAMHS were stratified by sex/gender. This approach is driven by previous research demonstrating a moderating effect of sex/gender on the association between school‐related variables and mental health outcomes (e.g., Bridges & Mumford, 2001).

We calculated absolute risk differences for accepted CAMHS referrals in those with high (>10% sessions missed) compared to low (≤10% sessions missed) levels of absence. In the main analysis, applying a >10% threshold for high absence, logistic regression was used to model the odds of receiving an accepted CAMHS referral by absence (high and low) for boys and girls separately, adjusted for all covariates (age, SEN provision, FSM eligibility, exclusion, and summer birth). Two sensitivity analyses were conducted. Firstly, an analysis excluding all those with CAMHS contact prior to the study period was conducted to reduce the impact of reverse causality issues. Secondly, an analysis including only those young people for whom there were data to confirm that they were living within the SLaM catchment area during the follow‐up period was conducted. Young people who moved out of the catchment area during the study period may have been accepted to CAMHS but in a different geographical area, thus underestimating the number of referrals to CAMHS within the whole cohort. In limiting the sample to those who were living within the catchment area, all 15‐year‐olds were excluded, as there were no data available on borough of residence for this group once they turned 16.

Additionally, different thresholds of ‘high absence’ were compared across two main metrics: sensitivity (i.e., the proportion of those who go on to have contact with mental health services who meet the high absence threshold) and positive predictive value (PPV; i.e., the proportion of those who meet the high absence threshold who go on to have contact with mental health services). We also report specificity (i.e., the proportion of those who did not go on to have contact with mental health services who did not meet the high absence threshold) and negative predictive value (NPV; i.e., the proportion of those who did not meet the high absence threshold who did not go on to have contact with mental health services).

## RESULTS

### Representativeness of analysis sample and missing data

Overall, 7.7% (*n* = 3998) of young people living in the SLaM catchment area and aged 11–15 at the start of the 2010/11 academic year (*n* = 51,924) did not have data available in the NPD for the 2010/11 academic year (7.1% of girls, 8.4% of boys) and therefore were not eligible for inclusion in the study.

There was a small amount of missing data for FSM status and SEN provision (*n* = 21 in each sex/gender); all variables were otherwise complete. Given the small proportion of missing data, analyses were conducted on a complete case basis.

### Descriptive statistics

The number of young people aged 11–15 at the start of the 2010/11 academic year, resident in the four SLaM catchment boroughs, and who had NPD data available, was 47,926. Of these, 1142 young people had an accepted referral to CAMHS within the follow‐up period.

**FIGURE 1:**
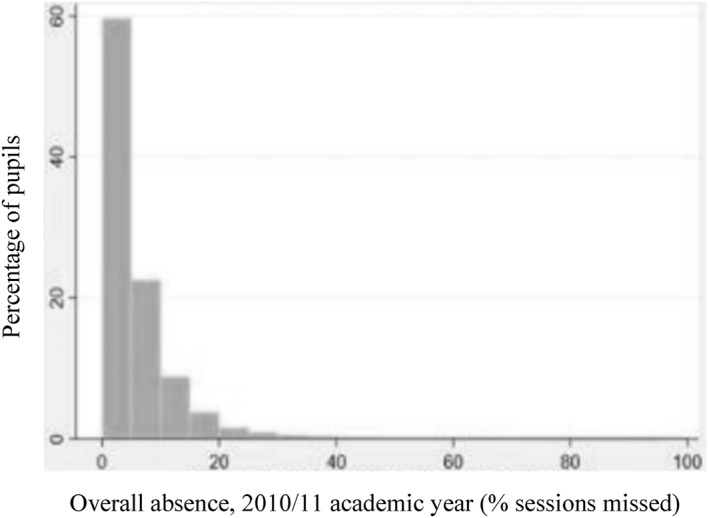
Distribution of overall absence calculated as proportion of half‐day sessions missed in the academic year 2010/11.

The distribution of overall absence can be seen in Figure 1. Absence data were positively skewed, with most young people having very low levels of absence.

Descriptive measures for absence—median, and percentage meeting a >10% threshold of high absence—are presented in Table 1; we did not generate means given the highly positive skew of absence rates (see Figure 1). Table 1 is stratified by sex/gender, first presented for the overall sample, and then by each of the covariates (age, SEN provision, FSM status, exclusion, and summer birth), the outcome variable (accepted CAMHS referral), and one additional variable for the sensitivity analysis (prior CAMHS contact). Information on the association between persistent absence (>10% sessions missed) and age can be found in the Supporting Information file (Table S1).

**TABLE 1:** Levels of absence for 11–15 year olds in 4 boroughs of South London by socio‐demographic, clinical, and educational factors during the 2010/11 academic year and by clinical outcomes during the 2011/12 academic year.

Baseline characteristics and CAMHS referrals	Girls			Boys		
	*N* (%)	Median absence (IQR)	% With >10% absence	*N* (%)	Median absence (IQR)	% With >10% absence
All	24,117 (100.0)	3.7 (1.3–7.8)	17.6	23,809 (100.0)	3.9 (1.4–7.9)	17.9
Age 11	4812 (20.0)	3.0 (1.3–6.3)	12.2	4770 (20.0)	3.3 (1.3–7.0)	13.8
Age 12	4801 (19.9)	3.7 (1.3–7.5)	15.3	4868 (20.5)	4.0 (1.6–7.6)	16.9
Age 13	4965 (20.6)	4.0 (1.6–8.1)	18.3	4939 (20.7)	4.0 (1.7–8.3)	19.4
Age 14	4752 (19.7)	4.0 (1.6–8.6)	20.5	4804 (20.2)	4.0 (1.3–8.3)	19.8
Age 15	4787 (19.9)	4.3 (1.6–8.9)	21.6	4428 (18.6)	4.0 (1.6–8.2)	19.7
SEN provision	6772 (28.1)	5.2 (2.0–10.8)	27.5	8899 (37.4)	5.0 (2.0–10.3)	26.1
No SEN provision	17,331 (71.9)	3.3 (1.3–6.8)	13.7	14,903 (62.6)	3.3 (1.3–6.7)	13.0
FSM eligible	6933 (28.8)	4.7 (2.0–9.7)	24.2	7014 (29.5)	4.9 (2.0–10.1)	25.4
Not FSM eligible	17,170 (71.2)	3.4 (1.3–7.0)	14.9	16,788 (70.5)	3.5 (1.3–7.1)	14.8
Exclusion	299 (1.2)	14 (8.3–26.0)	68.2	577 (2.4)	14.0 (7.7–24.3)	65.2
No exclusion	23,818 (98.8)	3.6 (1.3–7.6)	16.9	23,232 (97.6)	3.7 (1.3–7.6)	16.7
Summer born	8202 (34.0)	3.6 (1.3–7.6)	16.8	8216 (34.5)	3.9 (1.3–7.8)	17.8
Not summer born	15,915 (66.0)	3.9 (1.3–7.9)	18.0	15,593 (65.5)	3.9 (1.6–7.9)	18.0
CAMHS referral during follow‐up	636 (2.6)	8.1 (3.9–15.7)	43.2	506 (2.1)	7.0 (2.7–15.0)	37.2
No CAMHS referral during follow‐up	23,481 (97.4)	3.6 (1.3–7.6)	16.9	23,303 (97.9)	3.9 (1.4–7.7)	17.5
Prior CAMHS contact	1520 (6.3)	8.7 (3.6–17.8)	45.3	2114 (8.9)	7.8 (3.0–16.3)	41.8
No prior CAMHS contact	22,597 (93.8)	3.6 (1.3–7.3)	15.7	21,695 (91.1)	3.6 (1.3–7.3)	15.6

*Note*: Median absence represents percentage of available half‐day school sessions missed.

Abbreviations: CAMHS, Child and Adolescent Mental Health Services; FSM, Free School Meals; IQR, interquartile range; MCS, Millenium Cohort Study; SD, standard deviation; SEN, Special Educational Needs; SLaM, South London and Maudsley NHS Foundation Trust.

### Absolute risk differences

For girls, the percentage of pupils who received an accepted referral to CAMHS among those with high absence was 6.5% (95% CI 5.8–7.3), compared with 1.8% (95% CI 1.6–2.0) among those with low absence, with an absolute risk difference of 4.7% (95% CI 3.9–5.4). For boys, the percentage of pupils who received an accepted referral to CAMHS among those with high absence was 4.4% (95% CI 3.8–5.1), and 1.6% (95% CI 1.5–1.8) among those with low absence, with an absolute risk difference of 2.8% (95% CI 2.1–3.4).

### Logistic regression analyses

Table 2 shows the results of unadjusted and adjusted logistic regression models examining the association between high absence at the >10% threshold during the 2010/11 academic year and accepted referral to CAMHS during the follow‐up year.

**TABLE 2:** The association between high absence (>10% sessions missed) in 2010/11 and accepted referral to CAMHS in 2011/12 for adolescents aged 11–15 in 4 boroughs of South London.

	Girls		Boys	
	OR (95% CI)	aOR1 (95% CI)	OR (95% CI)	aOR1 (95% CI)
High absence	3.75^∗^^∗^^∗^ (3.20–4.41)	2.77^∗^^∗^^∗^ (2.33–3.30)	2.78^∗^^∗^^∗^ (2.32–3.35)	1.58^∗^^∗^^∗^ (1.29–1.95)
Age	1.08^∗^^∗^ (1.03–1.15)	1.05 (0.99–1.11)	0.95 (0.89–1.01)	0.97 (0.90–1.03)
FSM eligibility	1.31^∗^^∗^ (1.11–1.55)	0.99 (0.83–1.18)	1.62^∗^^∗^^∗^ (1.36–1.95)	1.11 (0.91–1.34)
SEN provision	2.32^∗^^∗^^∗^ (1.99–2.73)	1.70^∗^^∗^^∗^ (1.44–2.02)	3.68^∗^^∗^^∗^ (3.05–4.45)	2.75^∗^^∗^^∗^ (2.25–3.36)
Exclusion	13.88^∗^^∗^^∗^ (10.54–18.27)	7.00^∗^^∗^^∗^ (5.22–9.36)	12.31^∗^^∗^^∗^ (9.73–15.57)	6.67^∗^^∗^^∗^ (5.14–8.64)
Summer birth	1.04 (0.88–1.23)	1.06 (0.90–1.26)	0.98 (0.81–1.18)	0.95 (0.78–1.14)

*Note*: Missing data: FSM eligibility data: Girls *n* = 14, Boys *n* < 10; SEN provision data: Girls *n* = 14, Boys *n* < 10. Sample size for adjusted analyses: Girls *n* = 24,103, Boys *n* = 23,799–23,809 (exact number cannot be reported due to statistical disclosure rules). Total number of individuals in the sample irrespective of missing data in one or more variables: Girls *n* = 24,117, Boys *n* = 23,809.

Abbreviations: aOR, adjusted odds ratio; CAMHS, Child and Adolescent Mental Health Services; CI, confidence interval; FSM, Free School Meals; OR, unadjusted odds ratio; SEN, Special Educational Needs.

^∗^^∗^^∗^ *p* < 0.001, ^∗^^∗^ *p* < 0.01, ^∗^ *p* < 0.1.

Before adjusting for confounders, girls with high absence had 3.75 times the odds (95% CI 3.20–4.41) of an accepted referral to CAMHS within the following year than those with lower levels of absence. For boys, the odds ratio was 2.78 (95% CI 2.32–3.35). After adjusting for age, FSM eligibility, SEN provision, summer birth, and exclusion, the odds of an accepted referral to CAMHS within the following year in girls with >10% absence were 2.77 (95% CI 2.33–3.30) times higher than of those with lower levels of absence. For boys, the adjusted odds ratio was 1.58 (95% CI 1.29–1.95).

### Sensitivity analysis

A sensitivity analysis excluding those with previous contact with CAMHS was conducted. Unadjusted and adjusted associations between absence and CAMHS referrals were only slightly attenuated compared to the main analysis. For girls, the odds ratio of an accepted CAMHS referral for those with high compared with low absence was 3.12 (95% CI 2.51–3.87) and 2.54 (95% CI 2.01–3.20) after adjustment for covariates, compared with 3.75 (95% CI 3.20–4.41) and 2.77 (95% CI 2.33–3.30) in the main analysis, respectively. For boys, the unadjusted odds ratio was 2.59 (95% CI 1.96–3.42) and 2.54 (95% CI 2.01–3.20) after adjustment for covariates, compared with 2.78 (95% CI 2.32–3.35) and 1.58 (95% CI 1.29–1.95) in the main analysis respectively. See Table S2 for full details of the adjusted and unadjusted analyses, and an additional sensitivity analysis excluding those not resident in the SLaM catchment area at follow‐up (Table S3).

### Absence thresholds

The proportion of pupils who would be identified for additional investigation or support based on being in the ‘high absence’ category unsurprisingly increased as the threshold for absence lowered. Four fifths of pupils (80.9%) would require investigation with a threshold of >1% of available sessions missed, 40.3% if the threshold were >5%, 17.7% if it were >10%, 5.1% if >20%, 2.5% if >30%, 1.5% if >40% and 0.9% if >50%. Table 3 displays the performance of different absence thresholds in terms of their sensitivity, specificity, PPV and NPV of predicting later CAMHS contact. Sensitivity ranged from 3.9% at a threshold of >50% to 89.9% at a threshold of >1%, with the >10% absence threshold achieving a sensitivity of 40.5%. PPV ranged from 9.7% at the >50% threshold to 2.7% at the >1% threshold, and was 5.5% at the >10% absence threshold.

**TABLE 3:** Sensitivity, PPV, specificity, and NPV of identifying young people who will go on to have an accepted CAMHS referral by different thresholds for ‘high levels of absence’.

Threshold for ‘high absence’ (sessions missed)	Sensitivity (%)	PPV (%)	Specificity (%)	NPV (%)
>1%	89.8	2.7	19.4	98.7
>5%	65.2	3.9	60.3	98.6
>10%	40.5	5.5	82.8	98.3
>20%	17.7	8.3	95.2	97.9
>30%	9.8	9.3	97.7	97.8
>40%	6.2	9.9	98.6	97.7
>50%	3.9	9.7	99.1	97.7

*Note*: Excludes those with a prior history of CAMHS contact.

Abbreviations: CAMHS, Child and Adolescent Mental Health Services; NPV, negative predictive value; PPV, positive predictive value.

## DISCUSSION

This study investigated the association between school absence and subsequent accepted referrals to secondary care mental health services in a large sample of secondary school children in South London, UK, analysing these data in relation to a >10% absence threshold. This is a threshold for problematic absenteeism that has been applied in research and policy across multiple countries including the USA and Finland as well as the UK (e.g., Alanko et al., 2025; Kirksey, 2019).

Applying the >10% threshold for high absence, an almost fourfold increase in odds of accepted referral to CAMHS in the following year was seen in girls with high levels of absence and an almost threefold increase in odds in boys compared with low absence peers, before adjusting for confounders. Multivariable regression analyses yielded slightly attenuated associations between absence and accepted CAMHS referrals after adjustment for sociodemographic and educational variables. These data thus support previous findings from the USA and Canada showing a longitudinal relationship between school absence and mental health (Krause et al., 2025; Wood et al., 2012). The sensitivity analysis excluding those with previous contact with CAMHS replicated these findings.

There are a number of possible explanations for these findings. First, that school absence serves as a marker of underlying factors that increase the risk of mental illness, such as bullying (Bond et al., 2001) or parental mental health problems (Van Loon et al., 2014). Second, that there is a mechanism by which school absence contributes to the development of later mental health problems. This mechanism may operate through the impact of school absence on social isolation, which is known to contribute to poor mental health (Wright & Stickley, 2013) or through negatively impacting academic attainment (Rahman et al., 2018). It is also possible that in some cases, school absence is already being used as a marker of difficulties and that young people with poor attendance are being identified as having difficulties, thus increasing the opportunity for referrals to be made to mental health services.

Further research is required to explore the pathway between school absence and mental health, first by considering other unmeasured confounders which could help ascertain whether the relationship is causal, and second by testing possible mediators and mechanisms by which school absence could contribute to later mental health difficulties.

We compared the utility of the >10% absence threshold set by the UK government to other absence thresholds for their ability to identify young people at risk of requiring input from secondary care mental health services, focusing on sensitivity and PPV. Notably, the outcome variable of having an accepted CAMHS referral is a relatively rare outcome which serves as a proxy for more severe mental health problems; according to recent national surveys in England, 16.7% of young people aged 7–16 suffered from a probable mental disorder (Newlove‐Delgado et al., 2022), but only 25% of these were seen by specialist mental health services (Sadler et al., 2018). Although the PPV was low across all thresholds, this is less problematic for a rare and severe outcome than it would be for a more common and less severe outcome. On a practical level, the low PPV—that is, the ‘over‐prediction’ of risk in a school cohort—may still identify many in need of support who do not meet the threshold for clinical input. Hence, having a high sensitivity is more important. Here, the >10% threshold identified 17.7% of the population as having high levels of absence which in turn identified over 40% of young people who subsequently had an accepted referral to CAMHS. Although lower thresholds had greater sensitivity, these flagged proportions of the population as high risk which on a practical level may be too high for schools to manage were the threshold to be used as a marker of increased mental health risk.

Combined with the results of the regression, these results thus support the use of a threshold of >10% sessions missed as a predictor of increased risk, given that this threshold identifies a substantial but manageable proportion of the student population who are likely to benefit from support. That this threshold coincides with England's national absence policy definition of persistent absence would allow coherent and simple guidance to be issued to schools.

Although this study suggests that young people with high levels of absence have a greater risk of mental health problems than those with lower levels of absence, this is at population level, and there are of course young people at risk of mental illness with good school attendance who are also in need of support. For example, those experiencing problems at home may be more likely to attend school in order to receive support such as school‐based counselling. Conversely, not all young people with attendance problems will be at an increased risk of later mental health problems. If assumptions are made about the mental health of young people who miss school, this risks overmedicalisation and the potential to increase concerns about mental health unnecessarily. Therefore, it is essential that a holistic and personalised approach is taken when assessing attendance problems and underlying or associated difficulties and when offering support and intervention.

### Strengths and limitations

The use of routinely collected data enabled the longitudinal analysis of a very large general population sample, including the data of vulnerable populations who are sometimes excluded from traditional study designs (e.g., interviews and surveys; Perera et al., 2016). The use of routinely collected data also reduced the recall bias associated with traditional methods for measuring absence such as parental report.

A set of limitations to our conclusions pertains to characteristics of England's NPD. First, young people not attending state schools—for example, those who were home schooled, off‐rolled, or attending private schools—could not be included in the analysis. The relationship between school absence and mental health may be different for these groups; for example, young people with ‘unexplained exits’ from school enrolment may be more likely to be in contact with the social care system, eligible for FSM, and to have had high levels of absence from school (Hutchinson & Crenna‐Jennings, 2019). Second, there are several variables absent from the NPD which may influence the relationship between school absence and mental health problems, such as bullying (Bond et al., 2001), parental mental illness (Van Loon et al., 2014), and adverse childhood experiences (Bellis et al., 2018). This means that there is likely to be an element of residual confounding in the adjusted association between school absence and mental health reported in this study, limiting any causal assumptions that can be made. Another study in submission by our team (Epstein et al., in press) uses data from a nationally representative cohort study (The Millennium Cohort Study) linked to the NPD which contains data on additional variables mentioned above, and allows us to gain a better understanding of the direct association between school absence and mental health (although using a different measure for the mental health outcome). Furthermore, the binary coding of sex/gender in the NPD does not identify those with non‐binary genders, who may also experience a differential relationship between school absence and mental health. Finally, the way that data are presented in the NPD makes it difficult to distinguish absences from school due to exclusion from absences for other reasons. When data are entered by schools for the purpose of an NPD submission, there is a maximum number of sessions missed due to fixed term exclusions which can be included within the total number of sessions missed due to authorised absence. This means that to some extent, absences due to exclusion from school could have been double counted in some of the analysis models reported in this paper. This also means that when considering absence as a whole, this constitutes a broad grouping of reasons why a young person might be absent from school, with reasons ranging from illness to exclusion from school, which are qualitatively very different from each other.

In terms of properties of the data linkage, it is possible that linkage bias influenced the relationship between school absence and mental health through variables which were unavailable in the source data (Perera et al., 2016). There are also limitations with respect to the outcome measure used. As mentioned previously, this measure only captures those who were referred to, and accepted by secondary care mental health services and therefore likely to have more severe mental health problems. There are also young people who were referred to CAMHS but where the referral was rejected. We chose not to include this group within the definition of the outcome for this study in order to keep a more homogenous group given that the range of needs of those who were referred to but rejected by CAMHS could be extremely broad, and many are signposted to alternative agencies for support. Additionally, the sensitivity analysis only excluded those with previous contact with CAMHS. This does not exclude the possibility that some young people might have had mental health problems that were below the threshold for CAMHS input, potentially leading to reverse causality. Some young people are also likely to have accessed mental health support from other agencies, organisations, or individuals, including primary care and school‐based mental health services.

### Implications and future directions

These findings have important implications for health and education policy in England and internationally. The association between school absence and mental health is not always recognised at a policy level. In many countries including England, Norway, Sweden, and Denmark, school absence policy permits the issuance of sanctions on caregivers (Department for Education, 2024b; Skoubu et al., 2024). This punitive approach will have limited efficacy where the underlying reasons for school absence relate to psychosocial difficulties for the child or their family (Dalrymple, 2022). The association between school absence and subsequent mental health problems found here indicates the need for a more supportive approach to managing school absence, treating school absence as an opportunity to identify underlying problems and provide early intervention.

There are a number of ways in which these findings could be translated to changes in policy and practice. For example, when young people present with high levels of school absence, an assessment process should aim to understand the underlying factors contributing to that young person's attendance difficulties in order to provide the most appropriate intervention. Such factors may include individual and family factors, or influences related to the school environment or social difficulties at school (Hancock et al., 2018). Identification of and support for these underlying factors could prevent the development of later mental health problems and also result in improved school attendance. For example, if a young person's absence is primarily driven by anxiety, it may be that a treatment based on cognitive behavioural therapy (CBT) principles is the most suitable. Trials of such treatments have found that interventions resulted in improvements in both anxiety and school attendance (Heyne et al., 2011; King et al., 1998). On the other hand, if the attendance difficulties are primarily driven by issues relating to the school environment, changes to the school environment or policies may improve attendance and mental health outcomes for that young person, and potentially for many others (Ford et al., 2021; Patalay et al., 2020). There is, however, currently a limited evidence base for interventions to support young people with persistent absence, with most evidence generated in the United States of America, and limited evidence from Australia (Middleton et al., 2026). Therefore, one particular challenge for policymakers and intervention providers is to know how best to intervene based on knowledge about an individual's specific difficulties and reasons for school absence.

While there is also some evidence from Canada and Australia on the relationship between school absence and mental health (Hancock et al., 2018; Krause et al., 2025), not all has examined specific thresholds; future research should investigate whether the >10% absence threshold is useful for absence policy in countries and jurisdictions outside England. School‐level culture and policies, for example the wellbeing support available and whether absence is managed according to a supportive or punitive approach (Patalay et al., 2020), may also play an important role in the association between absence and mental health, and warrants further investigation.

Other future directions for exploring the relationship between school absence and mental health would be to investigate whether there is a dose‐response effect (i.e., longer duration of persistent absence yields a stronger association), to consider different trajectories or patterns of absence, and to use different lengths of follow‐up to indicate whether the relationship is acute or chronic. Moreover, the outcome variable employed here could not capture the many young people with mental health problems—sometimes severe—who have not been referred and/or accepted to CAMHS, as highlighted in recent national survey data. Hence, further research should also investigate the association between absence and mental health problems using different measures of mental health outcomes such as self‐ and parent‐report. Another study in submission by our team aims to do this by analysing self‐report data from a nationally representative cohort study linked to the NPD (Epstein et al., in press).

Future research should investigate whether the relationship between school absence and mental health observed here in data from 2010 to 2012 has since changed, especially in the context of the COVID‐19 pandemic. Levels of absence now are higher than prior to the pandemic in England (Department for Education, 2022) and around the world (e.g., Anders et al., 2026; Dee, 2024), and there is emerging evidence that school closures during the lockdowns were associated with poorer mental health in certain groups, but improved mental health in others (Soneson et al., 2023). We intend to replicate this study when newer data become available to evaluate this relationship in more recent years.

Finally, qualitative studies would be extremely valuable to understand the experiences of young people who struggle to attend school, as well as their families and school staff. For example, a previous study found that education professionals considered poor mental health and school absence to be related, but felt that school factors contributed less than individual, family, and peer factors to school absence (Finning et al., 2018). Future qualitative research could explore with all stakeholder groups, including young people and parents, their perceptions of how school absence might be related to later mental health and how best to support young people with school attendance problems in order to prevent later mental health problems.

## CONCLUSION

We linked routinely collected healthcare and education data in a longitudinal design to examine the association between school absence and subsequent accepted referrals to secondary care mental health services in a large sample of secondary school pupils in the UK. We found that young people who missed more than 10% of available school sessions over the course of an academic year were at increased risk of secondary care mental health service contact within the following year, and that this association remained after accounting for several socio‐demographic and educational factors. The >10% threshold of high absence provided moderate sensitivity (40%) for identifying those at risk of later secondary care mental health service contact while identifying a manageable proportion of the student population as high‐risk (18%). These findings have substantial implications for health and education policy at a national and international level, indicating that persistent school absence could be used to help identify young people at risk of mental health problems and offer early intervention. Further research is needed to better understand the nature of this association across other populations and with different measures of school absence and mental health, which may in turn illuminate the potential causal influence of school absence on mental health.

## AUTHOR CONTRIBUTIONS

**Sophie Epstein**: Conceptualization; investigation; writing—original draft; methodology; writing—review and editing; formal analysis; project administration; data curation; funding acquisition. **Zoë Firth**: Writing—review and editing. **Alice Wickersham**: Writing—review and editing; methodology. **Hitesh Shetty**: Data curation; software; resources; methodology; writing—review and editing. **Amelia Jewell**: Data curation; software; resources; methodology; writing—review and editing. **Rina Dutta**: Conceptualization; supervision; methodology; funding acquisition; writing—review and editing. **Craig Morgan**: Conceptualization; supervision; funding acquisition; methodology; writing—review and editing. **Tamsin Ford**: Conceptualization; supervision; funding acquisition; methodology; writing—review and editing. **Richard D. Hayes**: Methodology; writing—review and editing. **Johnny Downs**: Conceptualization; supervision; data curation; methodology; validation; funding acquisition; writing—review and editing.

## CONFLICT OF INTEREST STATEMENT

R.D.H. has received funding from The Cassel Hospital Charitable Trust, Janssen Research & Development LLC, and H. Lundbeck A/S. T.F.'s research group receives funding from Place2Be, a third sector organisation providing mental health training and interventions in UK schools. The remaining authors have declared that they have no competing or potential conflicts of interest.

## ETHICAL CONSIDERATIONS

The authors assert that all procedures contributing to this work comply with the ethical standards of the relevant national and institutional committees on human experimentation and with the Helsinki Declaration of 1975, as revised in 2013. All procedures involving human subjects/patients received research ethics committee (REC) approval as follows: ethics approval for analysis of data held in CRIS received REC approval for secondary analysis which has been updated every 5 years starting from 2008, most recently 2023 (Oxford REC C reference 08/H0606/71, 08/H0606/71 + 5, 18/SC/0372, 23/SC/0257) and formal SLaM Caldicott Guardian and Trust Executive approvals, as well as approval from the service‐user led CRIS Oversight Committee. Service users can opt out of their data being used for research. The linkage between CRIS and the NPD has ongoing research ethics approval from South Central—Oxford C Research Ethics Committee, most recently renewed in 2025 (25/SC/0229). Section 251 support to process confidential patient information for linkage without consent was provided by the Health Research Authority Confidentiality Advisory Group, most recently in 2020 (20/CAG/0068). As per the governance of service user data under the CRIS Oversight Committee, service users can opt out of their data being used for research.

## Supporting information

Tables S1–S3

## Data Availability

The data that support the findings of this study are available from Department for Education and the South London and Maudsley Biomedical Research Centre Clinical Record Interactive Search tool, which provides access to anonymised data derived from electronic medical records of the South London and Maudsley NHS Foundation Trust. Restrictions apply to the availability of these data, which were used under licence for this study. These data can only be accessed by permitted individuals from within a secure firewall. Permissions to access the data can be sought from the data owners.
