# Supplementary material for: School absence and mental health service referral: Cohort study of a South London data linkage
Source: JCPP Adv. 2026 Jul 9:e70144. Online ahead of print. doi: 10.1002/jcv2.70144 (PMC13351592; doi:10.1002/jcv2.70144)
Supplement: Supplementary file 1 — Tables S1–S3 [file JCV2-9999-e70144-s001.docx]

**School absence and mental health service referral:**

**Cohort study of a South London data linkage**

**Supporting Information**

**Contents**

[*Table S1: Association between persistent absence (>10% sessions missed) and age for girls and boys (OR and 95% CI)*](#_Table_S1:_Association)

[*Table S2 (reported sensitivity analysis): The association between high absence in 2010/11 and accepted referral to CAMHS in 2011/12 for adolescents aged 11-15 in 4 boroughs of South London excluding those with previous contact with CAMHS*](#_Supplementary_Table_3:)

[*Table S3 (additional sensitivity analysis): The association between high absence in 2010/11 and accepted referral to CAMHS in 2011/12 for adolescents aged 11-14 in 4 boroughs of South London excluding those not resident in the 4 boroughs of the SLaM catchment area during follow-up*](#_Supplementary_Table_4)

# Table S1: Association between persistent absence (>10% sessions missed) and age for girls and boys (OR and 95% CI)

|  | **OR (95% CI)** | |
| --- | --- | --- |
| **Age** | **Girls** | **Boys** |
| **11** | Baseline | Baseline |
| **12** | 1.30*  (1.16-1.46) | 1.27*  (1.13-1.41) |
| **13** | 1.62*  (1.44-1.81) | 1.50*  (1.35-1.67) |
| **14** | 1.86*  (1.66-2.07) | 1.54*  (1.38-1.72) |
| **15** | 1.98*  (1.77-2.21) | 1.52*  (1.37-1.70) |

*P < 0.001, OR = odds ratio, CI = confidence interval

# Table S2 (reported sensitivity analysis): The association between high absence in 2010/11 and accepted referral to CAMHS in 2011/12 for adolescents aged 11-15 in 4 boroughs of South London excluding those with previous contact with CAMHS

|  | | **Girls** | | **Boys** | |
| --- | --- | --- | --- | --- | --- |
|  | **OR**  (95% CI) | | **aOR**  (95% CI) | **OR**  (95% CI) | **aOR**  (95% CI) |
| **High absence** | 3.12***  (2.51-3.87) | | 2.54***  (2.01-3.20) | 2.59***  (1.96-3.42) | 1.68**  (1.23-2.29) |
| **Age** | 1.08*  (1.01-1.17) | | 1.06  (0.99-1.15) | 0.96  (0.88-1.06) | 0.98  (0.89-1.08) |
| **FSM eligibility** | 1.04  (0.83-1.31) | | 0.85  (0.67-1.08) | 1.29  (0.97-1.70) | 0.95  (0.71-1.28) |
| **SEN provision** | 1.52***  (1.22-1.89) | | 1.19  (0.95-1.50) | 2.51***  (1.93-3.26) | 2.02***  (1.53-2.66) |
| **Exclusion** | 34.12***  (22.11-52.64) | | 22.17***  (13.99-35.14) | 57.68***  (38.56-86.28) | 39.12***  (25.48-60.04) |
| **Summer birth** | 1.11  (0.89-1.37) | | 1.16  (0.93-1.44) | 0.90  (0.75-1.29) | 0.97  (0.73-1.28) |

Missing data: FSM eligibility data: Girls *n* = 13, Boys *n* < 10; SEN provision data: Girls *n* = 13, Boys *n* < 10

Sample size for adjusted analyses: Girls *n* = 22584, Boys *n* = 21685-21695 (exact number cannot be reported due to statistical disclosure rules)

Total number of individuals in the sample irrespective of missing data in one or more variables: Girls *n* = 22597, Boys *n* = 21695

OR = unadjusted odds ratio, aOR = adjusted odds ratio, CI = confidence interval, CAMHS = Child and Adolescent Mental Health Services, SEN = Special Educational Needs, FSM = Free School Meals

***P < 0.001 **P < 0.01 *P < 0.05

# Table S3 (additional sensitivity analysis): The association between high absence in 2010/11 and accepted referral to CAMHS in 2011/12 for adolescents aged 11-14 in 4 boroughs of South London excluding those not resident in the 4 boroughs of the SLaM catchment area during follow-up

|  | | **Girls** | | **Boys** | |
| --- | --- | --- | --- | --- | --- |
|  | **OR**  (95% CI) | | **aOR**  (95% CI) | **OR**  (95% CI) | **aOR**  (95% CI) |
| **Absence** | 4.22***  (3.51-5.08) | | 2.98***  (2.44-3.64) | 2.99***  (2.43-3.67) | 1.74  (1.38-2.19) |
| **Age** | 1.18***  (1.09-1.28) | | 1.11*  (1.02-1.21) | 0.96  (0.89-1.06) | 0.96  (0.88-1.06) |
| **FSM eligibility** | 1.40***  (1.16-1.68) | | 1.01  (0.83-1.23) | 1.76***  (1.44-2.15) | 1.22  (0.99-1.50) |
| **SEN provision** | 2.47***  (2.06-2.96) | | 1.77***  (1.46-2.15) | 3.80***  (3.07-4.70) | 2.84***  (2.27-3.55) |
| **Exclusion** | 16.51***  (12.18-22.38) | | 8.06***  (5.82-11.18) | 11.60***  (8.84-15.23) | 6.03***  (4.48-8.12) |
| **Summer birth** | 0.99  (0.82-1.20) | | 1.02  (0.84-1.24) | 0.99  (0.80-1.22) | 0.97  (0.78-1.20) |

Missing data: FSM eligibility data: Girls *n* < 10, Boys *n* = 0; SEN provision data: Girls *n* < 10, Boys *n =* 0

Sample size for adjusted analyses: Girls *n* = 18761-18771, (exact number cannot be reported due to statistical disclosure rules), Boys *n* = 18662

Total number of individuals in the sample irrespective of missing data in one or more variables: Girls *n* = 18771, Boys *n* = 18662

OR = unadjusted odds ratio, aOR = adjusted odds ratio, CI = confidence interval, CAMHS = Child and Adolescent Mental Health Services, SEN = Special Educational Needs, FSM = Free School Meals

***P < 0.001 **P < 0.01 *P < 0.05
